# Physicochemical properties and immune-enhancing activity of graded polysaccharides from the peels of stem lettuce (*Lactuca sativa*) by cascade membrane technology

**DOI:** 10.3389/fnut.2022.981099

**Published:** 2022-08-11

**Authors:** Xian-Guo Zou, Yu-Qin Cao, Jing-Jing Li, Xiao-Qian Guan, Ming Cai, Pei-Long Sun, Kai Yang

**Affiliations:** ^1^College of Food Science and Technology, Zhejiang University of Technology, Hangzhou, China; ^2^Key Laboratory of Food Macromolecular Resources Processing Technology Research, China National Light Industry, Zhejiang University of Technology, Hangzhou, China

**Keywords:** membrane technology, polysaccharides, stem lettuce, physicochemical properties, immune-enhancing activity

## Abstract

In this study, cascade membrane technology was applied to classify polysaccharides from the peels of stem lettuce (PPSLs), and three graded polysaccharides (PPSL100, PPSL10, and PPSL1) were obtained using ultrafiltration membranes of 100, 10, and 1 kDa in sequence. The physicochemical properties and immune-modulatory activity of three PPSLs fractions were analyzed and compared. Results showed that all three fractions have characteristic absorption peak of polysaccharides determined by FT-IR, and their monosaccharide composition only consisted of glucose determined by HPLC. PPSL10 had the highest contents of total sugar (88.09 ± 3.52%), uronic acid (2.55 ± 0.10%), and sulfate group (4.15 ± 0.20%). Besides, all three fractions exhibited immune-enhancing activities using RAW264.7 macrophages model, and PPSL10 was the best able to promote phagocytosis of neutral red and nitric oxide generation, which might relate to the high contents of above compositions and medium molecular weight (32 kDa). The findings indicated that PPSL10 could be developed as immune-modulator in the field of functional foods.

## Introduction

Polysaccharides are a kind of natural macromolecular substances mainly existing in plants, animals, and microorganisms. It is found that most polysaccharides are relatively harmless and would not produce obvious side effects (1). The health effects of plant polysaccharides are complex and multifaceted, including immune-regulatory, anti-inflammatory, anti-virus, anti-tumor activities, and hypoglycemic effect, which are closely related to their physiochemical characteristics, such as molecular weight (MW), monosaccharide composition, chemical composition, configuration, conformation, and glycoside bond (2–5). Therefore, the discovery of novel bioactive polysaccharides from the plant has been drawing significant attention.

Stem lettuce (*Lactuca sativa*), belonging to the composite family, is an annual or biennial herb. After long-term spread, it has become one of the most widely consumed vegetables in Asia, especially in China, and its Chinese name is “wosun” or “woju” (6). As a kind of homologous plant of medicine and food, it is rich in a variety of vitamins, minerals, folic acid, cellulose, and other nutrients, as well as functional components such as polysaccharides, phenols, terpenes (triterpenoids and sesquiterpenes; 7). For consumption, the leaves of stem lettuce are usually removed and the skins are peeled, and the inside soft translucent green core could be broiled or stewed as food. The peels of stem lettuce (PSLs) accounting for 30–40% of the raw material are discarded as waste from industry, resulting in environment stress and a great deal of resources waste. Actually, PSLs contained various nutritional and functional compounds, such as phenols, terpenoids, and polysaccharides. However, up to now, there is no knowledge about the physiochemical information of polysaccharides from PSLs, and whether they had immunoregulatory activities are unclear.

Macrophages are important subjects for exploring phagocytosis, cellular and molecular immunity, and they play crucial roles in the innate immune defense and adaptive immune responses (8). When the organism is stimulated by pathogenic organisms or injury, macrophages can produce large amounts of bioactive molecules, including nitric oxide (NO), various cytokines, and reactive oxygen species for defense (9). Therefore, macrophages are suitable cell models for evaluating the immunomodulatory activity of bioactive compounds. For example, Yang et al. (10) utilized the macrophages RAW264.7 cells as model to study the immune-enhancing ability of polysaccharide isolated from sea cucumber viscera, and found that the polysaccharide promoted the production of NO and cytokines.

The common method to separate polysaccharides from various sources is the usage of different concentrations of alcohol precipitation, requiring the depletion of a large amount of ethanol, and influencing the biological activities of polysaccharides to a certain extent (11). Membrane separation technology is a non-thermal, environmental friendly, solvent saving and effective method for classifying polysaccharides into some parts according to their different MWs. The application of membrane separation technology to prepare polysaccharides can be found in some published studies. Xu et al. (12) used ultrafiltration membrane with MWs of 10, 50, and 100 kDa in succession to obtain polysaccharides from camellia seed cake, and compared their characteristics and bioactivities. Additionally, cascade membrane technology was effectively used to grade polysaccharides extracted from *Ganoderma lucidum*, showing good antifatigue activity (13).

In this study, ultrafiltration membranes of the pore size 100, 10, and 1 kDa were selected to grade the aqueous extract from the PSLs, thus obtaining three polysaccharide fractions. The physiochemical characteristics of the graded polysaccharide from PSLs (PPSLs) were analyzed by the determination of the content of total sugar, reducing sugar, uronic acid, sulfate group, particle size, MWs, monosaccharide composition, and functional group. Besides, the immunomodulatory activity of PPSLs was evaluated by the measurement of cell viability, NO secretion and phagocytic activity using mouse macrophage RAW264.7 cells.

## Materials and methods

### Materials and chemicals

The powder of PSLs was provided by Ningbo Huikang Life Technology Co., Ltd (Ningbo, China). RAW264.7 macrophages were purchased from Cell Bank, Chinese Academy of Sciences (Shanghai, China). Testing kit of NO was obtained from Nanjing Jiancheng Bioengineering Institute (Nanjing, China). RPMI 1640 medium, penicillin, streptomycin, bovine serum albumin (BSA), lipopolysaccharide (LPS), fetal bovine serum and 3-(4,5-dimethylthiazol-2-yl)-2,5-diphenyltetrazolium bromide (MTT) were purchased from Gibco Life Technologies (Grand Island, NY, United States). Standards of mannose (Man, 99%), galactose (Gal, 99%), glucose (Glc, 99%), rhamnose (Rha, 99%), fucose (Fuc, 99%), and D-galacturonic acid (99%) were obtained from Sigma Aldrich (Shanghai, China). All other chemicals used in this study were from local company and of analytical grade.

### Extraction and purification of three polysaccharides from the peels of stem lettuce fractions

The powder of PSLs were extracted by hot water (95°C) for 4 h, and the extraction was repeated once. The extracting solution was centrifugated for 15 min, and the supernatant was collected, and then graded by ultrafiltration membranes (PES, Koch, United States) of 100, 10, and 1 kDa in sequence using the membrane separation equipment. Processes were carried out at a transmembrane pressure of 0.8 MPa and a revolving speed of 600 rpm. Three PPSLs fractions named PPSL100, PPSL10, and PPSL1 were obtained after the removal of protein using trichloroacetic acid and precipitation by 80% alcohol.

### Chemical components analysis of three polysaccharides from the peels of stem lettuce fractions

The total sugar contents of the PPSL1, PPSL10, and PPSL100 were determined by phenol sulfuric acid method (14) using glucose as the standard, while the reducing sugar contents of above three PPSLs fractions were analyzed by dinitrosalicylic acid method (15). The protein contents of three PPSLs fractions were assessed by Coomassie brilliant blue method (16) using BSA as the standard. The uronic acid contents in three PPSLs fractions were detected by m-hydroxybiphenyl method using D-galacturonic acid as the standard (17). The sulfate contents in three PPSLs fractions were determined with reference to the barium sulfate turbidimetric method using potassium sulfate as the standard (18).

### Molecular weight determination of three polysaccharides from the peels of stem lettuce fractions

The MW was determined by the high performance gel permeation chromatography (HPGPC; 19) equipped with three gel columns of TSKgel G3000PWXL (7.8 × 300 mm, 7 μm), TSKgel G4000PWXL (7.8 × 300 mm, 7 μm), and TSKgel G5000PWXL (7.8 × 300 mm, 10 μm) in series. The samples were completely dissolved in deionized water at the final concentration of 2.0 mg/mL, filtered through 0.22 μm microporous filter and then injected into the HPGPC system. The column was eluted with 0.02 M KH_2_PO_4_ at the flow rate of 0.6 mL/min and the column temperature was set at 35°C. The relative MW was calculated according to the calibration curve of ten standards with different MWs (3.62, 7, 9, 12.6, 20, 50, 126, 250, 496, and 1,000 kDa).

### Monosaccharide composition analysis of three polysaccharides from the peels of stem lettuce fractions

The monosaccharide compositions of PPSL1, PPSL10, and PPSL100 were analyzed using the pre-column derivatization HPLC method reported by Yang et al. (10) with minor modification. Briefly, the samples were hydrolyzed with 5 mL of trifluoroacetic acid (TFA) at 110°C for 4 h, and the hydrolysates were concentrated under vacuum with the addition of methanol until the TFA was completely removed. The solution of hydrolysate or monosaccharide standards (400 μL) was derivatived at 70°C for 1 h after mixing with 100 μL 1-Phenyl-3-methyl-5-pyrazolone (PMP, 0.5 M) and NaOH solution (100 μL, 0.3 M). After cooling, the derivatives were extracted by chloroform and analyzed by HPLC system (Agilent, United States) equipped with a C18 column (4.6 × 250 mm, 5 μm) and detected with a UV detector at 245 nm. The mobile phases were 0.05 M phosphate buffer (pH = 6.8, 82%) and acetonitrile (18%), and the injecting volume was 20 μL. The column was kept at 35°C and eluted at isocratic mode at a flow rate of 1 mL/min for 60 min.

### FT-IR analysis of three polysaccharides from the peels of stem lettuce fractions

The dried samples (5 mg) of PPSL1, PPSL10, or PPSL100 were fully mixed with KBr powder (100 mg), ground and pressed into particles for scanning in the spectral range of 4,000–400 cm^–1^.

### Particle size analysis of three polysaccharides from the peels of stem lettuce fractions

After full dissolution, the particle size of PPSL1, PPSL10, or PPSL100 was detected by dynamic light scattering (DLS) instrument (Zetasizer Nano series, Malvern) at 25°C. DLS analysis was performed at a scattering angle of 90°C. Each sample was determined for 10 s and three times, while each group ran for 11 times.

### Immune-modulatory effects of three polysaccharides from the peels of stem lettuce fractions

#### Cell culture

The RAW264.7 cell lines were cultured in RPMI-1640 medium containing 10% (v/v) FBS, 1% penicillin and streptomycin, and then maintained in a 5% CO_2_ constant temperature incubator at 37°C.

#### Cell viability assay

RAW264.7 cells in logarithmic growth period (1 × 10^4^ cells/well) were inoculated into 96-well plates for 24 h. Then, cells were treated with different concentrations of PPSL1, PPSL10, and PPSL100 (50, 100, 200, and 400 μg/mL), or LPS (1 μg/mL, positive control) for 24 h. After incubation, 20 μL MTT (5 mg/mL) solution was added to each well and incubated for another 4 h at 37°C in the dark. DMSO of 150 μL was added into each well and gently oscillated for 15 min to fully dissolve the formazan crystals. The absorbance was measured at 490 nm in each well by a microplate reader (Multiskan Go 1510, Thermo Fisher Scientific, United States).

#### Determination of nitric oxide production

RAW264.7 cells (1 × 10^4^ cells/well) were seeded into 96-well plates overnight, and then were treated with various concentrations of PPSL1, PPSL10, PPSL100 (50, 100, 200, and 400 μg/mL), and LPS for 24 h, respectively. After incubation, the supernatant was collected and the NO concentration was determined by Griess reagent kit with reference to the manufacturer’s instructions.

#### Phagocytosis assay

Neutral red is a weak alkaline which can be swallowed by living macrophage. Therefore, the effects of three PPSLs fractions on macrophage phagocytosis were measured by a neutral red phagocytosis assay (20). Briefly, RAW264.7 cells (1 × 10^4^ cells/well) were plated into 96-well plates and incubated for 24 h, and then treated with complete medium, different concentrations of PPSL1, PPSL10, or PPSL100 (50, 100, 200, and 400 μg/mL), or LPS for 24 h. Thereafter, 100 μL of neutral red solution (0.1%, w/w) was added to each well and incubated for 2 h. Cells were washed with PBS three times to remove the excess neutral red solution, and then added with 100 μL of the cell lysis buffer (ethanol: glacial acetic acid = 1: 1) to fully lyse the cells for 4 h. The absorbance at 540 nm was measured using the microplate reader.

### Statistical analysis

All experiments were repeated three times to minimize deviation, and data were presented as mean ± SD. Analyses of different comparisons between two groups were conducted using one-way ANOVA, followed by Turkey’s multiple range tests with the help of Origin 2021 software and Graphpad Prism 8.0. A value of *p* < 0.05 (*) and *p* < 0.01 (^**^) were regarded as statistically significance.

## Results and discussion

### Separation and molecular weights of three polysaccharides from the peels of stem lettuce fractions

The crude polysaccharides were obtained from the PSLs by hot-water extraction, and isolated using ultrafiltration membrane in different pore size (100, 10, and 1 kDa). Hence, three PPSLs fractions (PPSL1, PPSL10, and PPSL100) were collected after concentration and lyophilization, and their recovery rates were 4.39, 12.12, and 5.61%, respectively, (Table 1), indicating that PPSL10 was the main fraction of PPSLs.

**TABLE 1:** Chemical properties of three PPSLs fractions.

Indices	PPSL1	PPSL10	PPSL100
	
	(1–10 kDa)	(10–100 kDa)	(>100 kDa)
Yield (%)	4.39 ± 0.01c	12.12 ± 0.01a	5.61 ± 0.01b
Total sugar (%)	83.15 ± 4.09a	88.09 ± 3.52a	73.54 ± 3.17b
Reducing sugar (%)	13.65 ± 1.47a	5.63 ± 0.18b	3.66 ± 0.50c
Protein (%)	1.31 ± 0.01b	2.18 ± 0.09a	1.54 ± 0.05b
Uronic acid (%)	2.40 ± 0.09	2.55 ± 0.10	2.37 ± 0.06
Sulfate group (%)	2.67 ± 0.08b	4.15 ± 0.20a	2.59 ± 0.11b

The different letters (a, b, c) in the same line show significant difference (p < 0.05).

**FIGURE 1:**
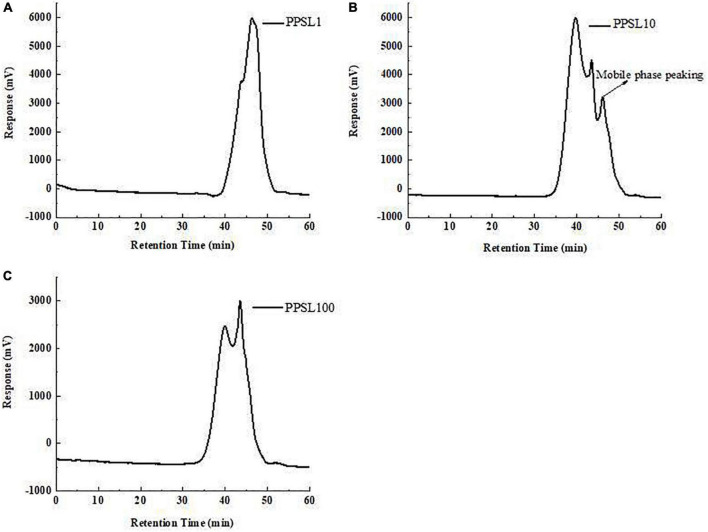
The average molecular weights of PPSL1 **(A)**, PPSL10 **(B)**, and PPSL100 **(C)** determined by HPGPC.

The relative MWs of three PPSLs fractions were determined by HPGPC using the dextran standards calibration curve. As shown in Figure 1, PPSL1 contained two components with MWs of 4.3 kDa (accounting for 31.29%) and 1.8 kDa (accounting for 68.71%). PPSL10 was composed of a main peak accounting for 77.81% and an adjacent peak, and the MW of the main component was 32 kDa. PPSL100 also contained two peaks with MWs of 129 kDa and 6.1 kDa, accounting for 60.15 and 39.85%, respectively. The small molecular polysaccharides (6.1 kDa) appeared in the PPSL100 fraction might be due to the uncomplete gradation of polysaccharides in the intercepted solution.

### Chemical composition of three polysaccharides from the peels of stem lettuce fractions

As listed in Table 1, three PPSLs fractions contained different proportions of total sugar, uronic acid, and sulfate groups. PPSL10 had the highest contents of total sugar (88.09 ± 3.52%) and sulfate group (4.15 ± 0.20%), followed by PPSL1 and PPSL100. Besides, the uronic acid content in PPSL10 was slightly higher than that in other two fractions. However, the content of reducing sugar in the PPSL1 was the maximum (13.65 ± 1.47%), and that in PPSL100 was the lowest (3.66 ± 0.50%). The protein contents of three PPSLs fractions were below 3%, suggesting that most proteins were removed during isolation. Similar to our findings, Yang et al. (10) also reported the presence of uronic acid and sulfate groups in the polysaccharides isolated from sea cucumber viscera. Besides, the types of chemical components in the PPSLs from our study were almost consistent with that found in the core of stem lettuce from the study of Nie et al. (21), but showing discrepancy in contents. The difference in contents might result from different extraction parts of stem lettuce or preparation methods.

### Monosaccharide composition analysis of three polysaccharides from the peels of stem lettuce fractions

**FIGURE 2:**
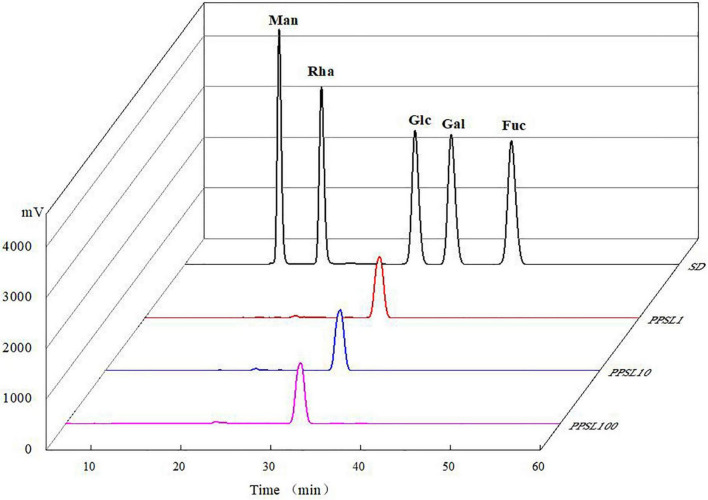
Monosaccharide compositions of mixed standards, PPSL1, PPSL10, and PPSL100.

After hydrolyzing by TFA and derivatizating by PMP, the monosaccharide compositions of PPSL1, PPSL10, and PPSL100 were analyzed through HPLC technology. As shown in Figure 2, the peaks of mixed standards were Man, Rha, Glc, Gal, and Fuc according to the order of their retention time. In all three fractions only one peak appeared on the recording graph, and it was identified as glucose when compared with the mixed standards. In accord with our result, Ling et al. (22) reported that the polysaccharide extracted from *Mactra veneriformis* only consisted of D-glucose residue. However, our result was different from several studies. Ji et al. (23) obtained a medium MW polysaccharide from *Ziziphus Jujuba* Mill. (PZMP4), and found that it consisted of rhamnose, arabinose, mannose, glucose, galactose, and galacturonic acid with the molar ratio of 2.32: 2.21: 0.22: 0.88: 2.08: 8.83. Besides, they obtained a high MW polysaccharide from *Ziziphus Jujuba* cv. Muzao (PZMP2-1), and found that it was composed of rhamnose, arabinose, galactose, and galacturonic acid at a ratio of 0.84: 5.88: 0.31: 0.12 (24). Nie et al. (25) obtained two fractions of SLP-1 and SLP-2 from the core of stem lettuce using 75% ethanol extraction, and found that the monosaccharide composition of SLP-1 was Man, Rha, GalA, Gal, and Ara in molar percentages of 3.6: 3.2: 17.6: 41.7: 33.9, and the monosaccharide composition of SLP-2 was Man, Rha, GalA, Gal, and Ara in molar percentages of 11.5: 1.5: 69.5: 9.3: 8.2. This might be due to different varieties of stem lettuce or different extraction parts or extraction method.

### FT-IR spectroscopy analysis of three polysaccharides from the peels of stem lettuce fractions

**FIGURE 3:**
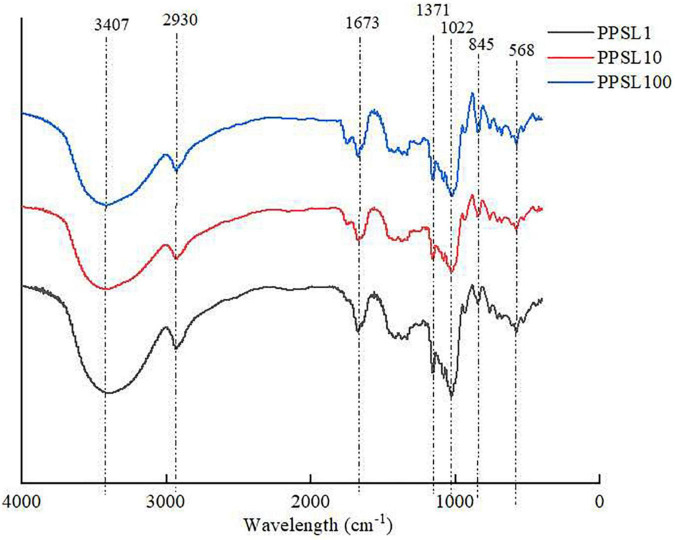
FT-IR spectrum of PPSL1, PPSL10, and PPSL100.

IR spectroscopy is a useful and reliable technique for the identification of characteristic functional groups and glycosides in a polysaccharide (26). As presented in Figure 3, three PPSLs fractions (PPSL1, PPSL10, and PPSL100) showed similar characteristic bands in the FT-IR spectrum. The broad and strong absorption at 3,407 cm^–1^ are characteristic of the O-H stretching vibrations of hydroxyl groups, and the weak signal around 2,930 cm^–1^ was assigned to the stretching vibration of C-H single bond (27). The band at 1,673 cm^–1^ might represent the asymmetric stretching vibration of C = O, indicating the presence of uronic acid (28). Besides, the signals at 1,371, 845, and 568 cm^–1^ revealed the presence of sulfate in three PPSLs fractions (10). Specifically, the absorption peak at 1,371 cm^–1^ corresponded to the symmetric stretching vibration of S = O, which at 568 cm^–1^ reflected the stretching vibration of S-O. The peak at 845 cm^–1^ was caused by the stretching of the symmetric C-O-S of sulfate. The presence of characteristic functional group such as uronic acid and sulfate in three PPSLs fractions was in agreement with their chemical compositions.

### Particle size observation of three polysaccharides from the peels of stem lettuce fractions

**FIGURE 4:**
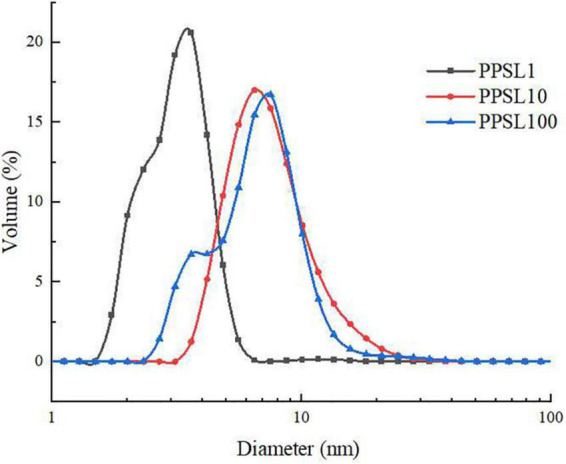
Particle size diagram of PPSL1, PPSL10, and PPSL100.

Dynamic light scattering was used to detect the particle size distribution of three PPSLs fractions. As shown in Figure 4, PPSL1, PPSL10, and PPSL100 had different distribution range of particle size. PPSL1 had the minimum average particle size (below 10 nm), followed by PPSL10 and PPSL100, which is consistent with the separation membrane pore size.

### Immune-modulatory activities of three polysaccharides from the peels of stem lettuce fractions

#### Effects of three polysaccharides from the peels of stem lettuce fractions on macrophage viability and phagocytosis

**FIGURE 5:**
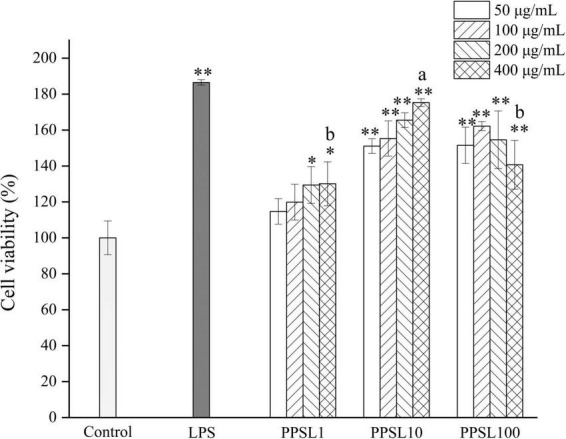
Effects of different concentrations of three PPSLs fractions (50, 100, 200, and 400 μg/mL) on cell viabilities of RAW264.7 macrophages. *p < 0.05, **p < 0.01 vs. control group. The different letters (a, b) showed significant difference in the same concentration among three PPSLs fractions (*p* < 0.05).

Macrophage activation is an essential process to advance the body’s innate immune defense capabilities and maintain homeostasis. In this study, the immunological activities of PPSL1, PPSL10, and PPSL100 were examined using RAW264.7 murine macrophages. To first investigate the toxicity of three PPSLs fractions, MTT assay was used to examine cell proliferation. As shown in Figure 5, LPS (positive control) and three PPSLs fractions at indicated concentrations (50–400 μg/mL) exhibited no cytotoxic effect on RAW264.7 cells and increased their proliferation. Especially, the PPSL10 showed the most obvious cell proliferation effect. At the PPSL10 concentration of 400 μg/mL, cell viability reached to the maximum value, enhancing cell growth by 70.20 ± 2.10% as compared to the blank group.

**FIGURE 6:**
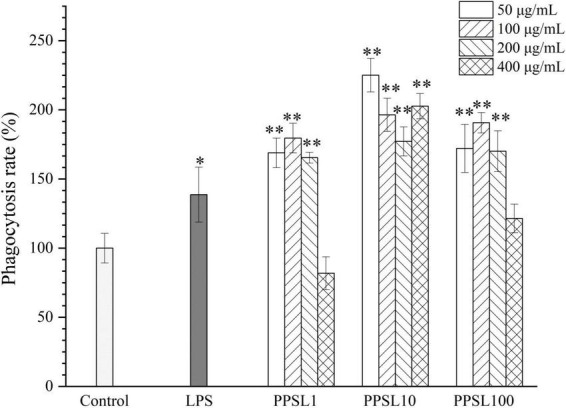
Effects of different concentrations of three PPSLs fractions (50, 100, 200, and 400 μg/mL) on phagocytic activities in RAW264.7 macrophages. *p < 0.05, ^**^p < 0.01 vs. control group.

Phagocytosis is a marked feature of activated macrophages and represents the most pivotal process in the immune response to exogenous stimulus (29). In this study, the phagocytic effect of RAW264.7 cells treated with different concentrations of three PPSLs fractions (50–400 μg/mL) were detected by the neutral red uptake assay. As shown in Figure 6, PPSL1 and PPSL100 at the concentrations range of 50–200 μg/mL significantly increased the phagocytic rate, however, they showed no significant change in the phagocytic rate when their concentrations reached to 400 μg/mL as compared to the blank group. Notably, PPSL10 at tested concentrations of 50–400 μg/mL showed the strongest phagocytic ability among three PPSLs fractions. This is consistent with the findings of Shen et al. (30), who reported that the polysaccharides isolated from the blossoms of *Hibiscus sabdariffa* activated macrophages through MAPK and NF-κB signaling pathways.

#### Effect of three polysaccharides from the peels of stem lettuce fractions on nitric oxide secretion

**FIGURE 7:**
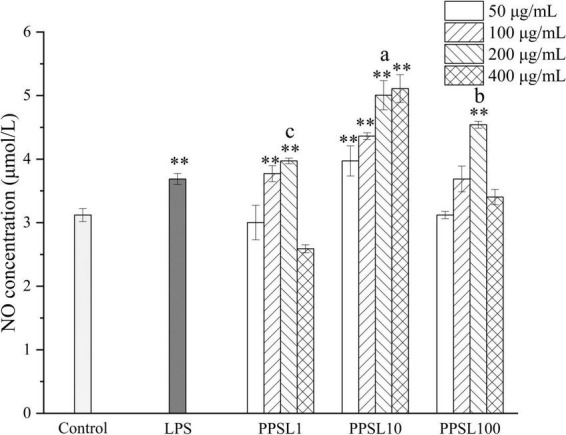
Effects of different concentrations of three PPSLs fractions (50, 100, 200, and 400 μg/mL) on NO production of RAW264.7 macrophages. **p < 0.01 vs. control group. The different letters (a, b, c) showed significant difference in the same concentration among three PPSLs fractions (*p* < 0.05).

Activated macrophages generate diverse cytokines and factors, such as NO, TNF-α, IL-6, IL-1β, and PGE2, which are crucial in immune responses (31). In particular, NO, an important signal transduction gaseous molecule involved in the host defenses against microorganisms and apoptotic cells can strengthen the phagocytosis of macrophages (32). Hence, we detected the NO production after treating macrophage with PPSL10-2 (50–400 μg/mL) using the Griess method. As shown in Figure 7, LPS and PPSL10 at indicated concentrations range significantly (*p* < 0.01) promoted the release of NO. However, PPSL1 at concentrations of 100 and 200 μg/mL, and PPSL100 only at concentration of 200 μg/mL significantly (*p* < 0.01) elevated the level of NO production. Overall, the results indicated that PPSL10 was the best to enhance NO generation, which were in accord with those of phagocytic activity.

Taken together, PPSL10 had strong immune-enhancing activities, followed by PPSL1 and PPSL100. The differences in biological activities of three PPSLs fraction were related to their chemical composition and MWs. Wu et al. (33–34) found that the immunomodulatory activity and microbial fermentation characteristics of pectic-polysaccharide extracted from okra were closely related to its degree of branching and MW. Numerous studies revealed that the immunological and antioxidant activities of polysaccharides were based on the content of sulfate group or acid (9, 35, 36). In this study, the sulfate group and uronic acid contents in PPSL10 fraction were the highest (Table 1). In addition, the MWs among three PPSLs fractions were quite different, PPSL10 had a medium MW (Figure 1). Supporting our results, Kakutani et al. (37) reported that glycogens owning MW more than 10,000 K could not activate macrophages, whereas glycogens owning medium MWs (5,000 K and 6, 500 K) exhibited strong immune-modulatory activities. According to results from the study of Shen et al. (30), polysaccharides from *Hibiscus sabdariffa* Linn. *Via* and green alga *Ulva intestinalis* possessing medium MW were reported to have better immunostimulating capabilities. Besides, Leiro et al. (38) obtained a sulfated polysaccharide from *Ulva rigida*, and found that the synthesis of NO in macrophages was proportionally correlated with the sulfate content. Therefore, the relative high contents of uronic acid and sulfate group as well as medium MW might partially explain the strong immune enhancement ability of PPSL10. Future study will focuse on the separation, characterization of PPSL10 fractions to further illuminate structure-activity relationship.

## Conclusion

Peels of stem lettuce are regarded as waste during stem lettuce processing but the peels are actually good raw materials of polysaccharides. Membrane technology with different pore size can be used to grade polysaccharides on the basis of their MW, which is of great importance to differentiate the physicochemical properties and bioactivities of polysaccharides. Therefore, in this study, cascade membrane technology was applied to classify the PPSLs into three fractions. The physicochemical characteristics and immune-stimulating activity of three PPSLs fractions were investigated and compared. Resultantly, all three fractions were found to contain only glucose and exhibit immune-enhancing activities through increasing phagocytosis and NO production, especially for PPSL10, which might be due to its high uronic acid and sulfate group as well as medium MW. Our findings would shine light on the added value of stem lettuce, providing a new direction for the utilization and development of stem lettuce peel. Further study will focus on improving the purity and illustrating the structure and bioactivity of PPSL10.

## Data availability statement

The original contributions presented in this study are included in the article/supplementary material, further inquiries can be directed to the corresponding authors.

## Author contributions

X-GZ: conceptualization, writing—review and editing, and visualization. Y-QC: formal analysis, software, and writing—original draft. J-JL: investigation and methodology. X-QG: software and methodology. MC: validation and data curation. P-LS: supervision and project administration. KY: conceptualization, methodology, and writing—review and editing. All authors contributed to the article and approved the submitted version.
